# The Use of a Non-Absorbable Membrane as an Occlusive Barrier for Alveolar Ridge Preservation: A One Year Follow-Up Prospective Cohort Study

**DOI:** 10.3390/antibiotics9030110

**Published:** 2020-03-03

**Authors:** Piero Papi, Bianca Di Murro, Marco Tromba, Pier Carmine Passarelli, Antonio D’Addona, Giorgio Pompa

**Affiliations:** 1 Department of Oral and Maxillo-Facial Sciences, “Sapienza” University of Rome, 00161 Rome, Italy; bianca.dimurro@uniroma1.it (B.D.M.); marcotrombadr@gmail.com (M.T.); giorgio.pompa@uniroma1.it (G.P.); 2 Division of Oral Surgery and Implantology, Department of Head and Neck, Oral Surgery, and Implantology Unit, Institute of Clinical Dentistry, Catholic University of Sacred Hearth, Fondazione Policlinico Universitario Gemelli, 00168 Rome, Italy; piercarminepassarelli@hotmail.it (P.C.P.); antonio.daddona@gmail.com (A.D.)

**Keywords:** guided bone regeneration, dense-polytetrafluoroethylene (d-ptfe), alveolar ridge preservation, Non-absorbable membrane, biomaterials

## Abstract

The aims of this study were to obtain preliminary data and test the clinical efficacy of a novel nonporous dense-polytetrafluoroethylene (d-PTFE) membrane (permamem^®^, botiss) in alveolar ridge preservation (ARP) procedures with a flapless approach. A traumatic extraction was performed in the premolar maxillary area, and a d-PTFE membrane was used to seal the alveolar cavity: no biomaterial was used to graft the socket and the membrane was left intentionally exposed and stabilized with sutures. The membrane was removed after four weeks and dental implants were placed four months after the procedure. The primary outcome variables were defined as the dimensional changes in the ridge width and height after four months. A total of 15 patients were enrolled in this study. The mean width of the alveolar cavity was 8.9 ± 1.1 mm immediately after tooth extraction, while four months later a mean reduction of 1.75 mm was experienced. A mean vertical reduction of 0.9 ± 0.42 mm on the buccal aspect and 0.6 ± 0.23 mm on the palatal aspect were recorded at implant placement. Within the limitations of this study, the d-PTFE membrane proved to be effective in alveolar ridge preservation, with the outcomes of the regeneration not affected by the complete exposure of this biomaterial.

## 1. Introduction 

The alveolar process is a tooth-dependent structure [1,2]; therefore, dimensional and morphologic alterations occur after the extraction, with significant bone remodeling within the first six months [3,4,5]. The bone reabsorption pattern is widely influenced by tooth site, with volumetric changes occurring more severely in the anterior sector [6,7,8]. According to Chappuis et al. [9], the facial bone wall thickness is the main factor affecting bone remodeling in incisors and premolars in the upper maxilla, with a vertical bone reduction up to 7.5 mm. These three-dimensional alterations may affect the outcomes of future implant supported restorations, leading to aesthetic compromises [10,11]. 

Furthermore, a significant reduction in the keratinized mucosa width (KMW) can be observed after tooth extraction [12,13], with the possible need to perform additional soft tissue augmentation procedures [14], due to the positive role of KMW >2 mm in promoting peri-implant health [15,16,17] and preventing implant-biologic complications [18,19].

Over the years, different techniques and materials have been proposed to preserve ridge dimensions, minimizing hard and soft tissue alterations following tooth extractions [20,21,22].

Several studies [23,24] have shown that a flapless approach has been proven to reduce healing times, discomfort, and inflammation, preserving the periosteum and, therefore, blood supply to the underlying bone. According to recent systematic reviews [25,26,27], alveolar ridge preservation (ARP) procedures are effective in minimizing both horizontal and vertical hard tissue dimensional loss compared to spontaneous healing alone, with insufficient evidence to determine whether there is a superior approach.

Several biomaterials are currently used for grafting the extraction socket, with or without the combination of a reabsorbable barrier membrane [28,29]. A certain amount of time is required for graft maturation and surgical re-entry, and implant placement is usually performed after a minimum of 16 weeks, with the majority of bone substitutes requiring at least six months of healing [30,31]. 

The use of collagen membranes usually requires a flap procedure with a tension-free closure in order to minimize the risk of membrane exposure, which may jeopardize the duration of barrier function and the amount of bone fill [32,33].

Non-absorbable membranes in expanded-polytetrafluoroethylene (e-PTFE) have been successfully used for guided bone regeneration (GBR) procedures in dentistry for years [34,35]; however, their main disadvantage is that membrane exposure can lead to bacterial infection, with a subsequent early removal of the membrane [36].

Therefore, nonporous dense-polytetrafluoroethylene (d-PTFE) barrier membranes have become widespread [37,38]. Due to their reduced pore size and minor bacterial infiltration, membranes can be left completely exposed in the oral cavity, without a detrimental effect on final outcome of the regeneration.

Furthermore, several studies [39,40,41] have shown that ridge preservation procedures can be performed using d-PTFE membranes without primary closure, with an open healing approach, allowing a significant increase of KMW due to the absence of vertical releasing incisions displacing the muco-gingival junction (MGJ) and maintenance of the blood clot, with no need of bone grafting in the socket. 

The aims of this study were to obtain preliminary data and test the clinical effectiveness and efficacy of a novel d-PTFE membrane (permamem^®^, botiss, Berlin, Germany) in alveolar ridge preservation procedures with a flapless approach.

## 2. Results

A total of 15 patients were enrolled in this study; they were either males (9) or females (6), with a mean age of 49.4 ± 10.4 years (range: 35–67 years) (Table 1).

Reasons for tooth extraction varied, and included root fracture (4), caries (3), root resorption (1), or endodontic failure (7).

After tooth extraction, healing was uneventful and membranes were removed non-surgically without anesthesia after four weeks. No bacterial infection was detected in any of the cases treated.

**Chart 1:**
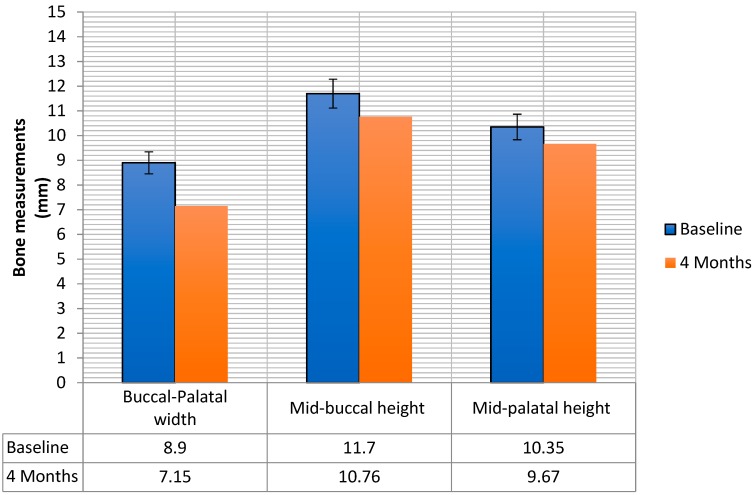
Dimensional changes in the ridge width and height at four months after the procedure.

The mean width of the alveolar cavity was 8.9 ± 1.1 mm immediately after tooth extraction, while four months after the procedure, a mean reduction of 1.75 mm was experienced, with mean values of buccal-palatal width of 7.15 ± 1.3 mm and no statistically significant intragroup differences (*p* > 0.05). Mean values of mid-buccal height and mid-palatal height at baseline were 11.7 ± 1.1 mm and 10.35 ± 1.1 mm, respectively (Chart 1).

A mean vertical reduction of 0.9 ± 0.42 mm on the buccal aspect and 0.6 ± 0.23 mm on the palatal aspect was recorded at implant placement (*p* > 0.05) (Chart 1).

As for secondary variables, the mean KMW value was 2.37 ± 0.89 mm at baseline, while four months later, an average of 3.5 ± 1.26 mm was measured.

After alveolar ridge preservation (ARP) procedures, either narrow (3.75 mm) or standard (4.5 mm) dental implants were placed, with lengths varying from 10 to 12 mm (Table 1).

All implants inserted showed a maximum insertion torque greater than 35 Ncm, with a mean value of 41.9 ± 5.02 (range: 36–50 Ncm), while mean Implant Stability Quotient (ISQ) values were 68.6 ± 3.56 (range: 63–75) at baseline and 69.5 ± 2.63 (range: 67–76) after one year of functional loading.

As for peri-implant clinical and radiographic parameters, the mean probing pocket depths (PPD) values were 2.55 ± 0.65 mm after one year with no significant increase (*p* > 0.05) from PPD-baseline (2.25 ± 0.45 mm). Mean marginal bone loss (MBL) was 0.22 ± 0.13 mm, while bleeding on probing was negative on all implants.

## 3. Discussion

According to several authors [39,40,41], non-absorbable membranes have proven to be effective in alveolar ridge preservation procedures, even with no biomaterial filling in the sockets.

According to Laurito et al. [41], bacterial contamination was not observed in the internal surface of d-PTFE membranes left exposed for four weeks, although plaque accumulation was present on the external surface. Thus, the small pore size (less than 0.2 microns) prevents bacterial penetration and, therefore, avoids the necessity of obtaining a primary closure, allowing complete exposure of the membrane, with no need for large flaps or vertical releasing incisions.

Furthermore, the antibacterial properties of the membrane lower the need for systemic antibiotic therapy. Only prophylactic antibiotics were prescribed in this study, with no need for full antibiotic coverage. 

This could be explained by the fact that the average bacteria size is larger than 0.2 microns and they cannot penetrate in the alveolar cavity, reducing infection risk at extraction site.

To the best of the authors’ knowledge, this is the first clinical study with this novel d-PTFE membrane, characterized by an extremely thin structure (~0.08 mm).

The membrane was used as an occlusive barrier for maintaining the integrity of the clot and was stabilized on the residual bony walls. 

The absence of a graft material inside the socket is an important advantage, since there are no residual graft particles which can impair implant survival, and there is a significant shortening of treatment times because there is no need to wait more than four months for surgical re-entry [30,31].

No patient presented signs of inflammation or lost the membrane before the scheduled appointment, and removal was performed after four weeks without injury to the underlining tissues or discomfort for patients. According to several authors [39,40,41], histologic analysis of sockets treated with exposed d-PTFE membranes revealed the formation of a dense structure of connective tissue at 28 days, protecting the underlying bone with no bacterial infiltration. After this interval, the membrane tended to lose stability due to the apical migration of the flap. The results in the present study demonstrated that the membrane was effective in limiting bone reduction after a tooth extraction. Hence, mean horizontal bone loss after four months was 1.75 mm, while a recent systematic review [42] reported a mean loss of 2.79 mm after the same interval for flapless extractions with spontaneous healing. Mean mid-buccal vertical height loss was 0.9 mm, compared to the median 1.74 mm loss reported by Jambhekar et al. [42] for the studies with no intervention. 

Regarding keratinized mucosa width, a mean gain of 1.1 mm was obtained in this study after four months, a result in accordance with Avila-Ortiz et al. [43], who described only slightly lower gains of KMW (<1 mm) in a randomized control clinical trial for three different ARP approaches using PTFE membranes. 

In contrast to our findings, Mandarino et al. [44] reported a mean keratinized gingival gain of 4.30 ± 1.20 mm after the use of intentionally exposed d-PTFE membranes in tooth extraction, with Barboza et al. [45], showing a mean increase of 7.06 ± 2.63 mm.

However, in the above-mentioned articles [44,45], the authors measured keratinized mucosa (KM) from the buccal MGJ to the lingual MGJ, while in the present study, and in Avila-Ortiz et al. [43], KM was assessed from the buccal MGJ to the gingival margin.

Non-absorbable d-PTFE membranes have proven to be effective and extremely predictable in increasing the zone of keratinized tissue [41,43,44,45]. Releasing incisions are not required to advance the flap, therefore the MGJ is not displaced and KM is preserved either on the buccal and lingual aspects.

Another interesting finding obtained was the high primary stability of dental implants installed: they reached all values of insertion torque greater than 35 Ncm, with also ISQ values > 65 at baseline and after one year of functional loading.

A possible explanation could be the macrogeometry of the implant used: with a self-cutting and self-tapping design, the novel implant was capable of reaching an excellent primary stability in all clinical situations. According to Deli et al. [46], ISQ values in the maxillary premolar area were statistically significantly (*p* > 0.05) higher in regenerated sockets compared to healed sites.

Just a few articles [47,48] have evaluated the influence of alveolar ridge preservation on implant stability, but to the best of the authors’ knowledge there are no studies reporting data on the use of d-ptfe membranes alone. 

The main limitations of this study included the absence of a control group and the small sample size; however, this is the first article providing clinical data on this novel membrane. 

Alveolar ridge preservation procedures were performed in the same area (upper maxillary premolar) for all patients included, while the same implant was used in all cases and surgeries were carried out by the same operator. This could constitute another major limitation, since the reproducibility of this technique and, therefore, its results could be subjected to learning curve and operator dependency. Randomized controlled clinical trials are needed to confirm properties of this material.

## 4. Conclusions

Within the limitations of this study, the novel d-PTFE membrane has proven to be effective in alveolar ridge preservation procedures, even intentionally left exposed and with no biomaterial filling in the sockets. Further studies, with a control group and a larger sample, are needed to confirm our findings.

## 5. Material and Methods

### 5.1. Study Design and Patient Selection

To address the research purpose, the authors developed and implemented this case series, conducted at the Departments of Oral and Maxillo-Facial Sciences, at “Sapienza” University of Rome.

From March 2018 to June 2018, all subjects referred for tooth extraction to the Oral Surgery Unit, Policlinico Umberto I, “Sapienza” University of Rome, were consecutively evaluated. 

In order to be included in the study, patients had to meet the following inclusion and exclusion criteria: need for a tooth extraction in the premolar maxillary region (FDI position 14-15–24-25), presence of natural adjacent teeth, integrity of the four bony walls, good oral hygiene (full mouth plaque score and full mouth bleeding score < 25%), absence of uncontrolled systemic diseases, non-smokers (<10 cigarettes per day), and no signs of local inflammation.

Each patient received detailed descriptions of the study protocol and all subjects signed the inform consent form and gave written approval to be included in the study population, according to the latest version of the World Medical Declaration of Helsinki (2013). The study was approved by the Institutional Review Board of the Department of Oral and Maxillo-Facial Sciences at “Sapienza” University of Rome.

### 5.2. Clinical Parameters 

The primary outcome variables were defined as the dimensional changes in the ridge width and height at four months after the procedure. 

At the time of the surgery, immediately after tooth extraction, direct measurements of the width of the alveolar cavity, from the mid-crest of the buccal wall to the mid-crest of the palatal wall were taken with a calibrated periodontal probe to the nearest mm (PCP-Unc 15, Hu-Friedy^®^, Chicago, Illinois, USA) by the same operator (BDM). The height of the buccal and palatal bony walls was measured vertically from a line connecting the cemento-enamel junctions (CEJs) of adjacent teeth at the mesio-distal midpoint between those adjacent teeth.

The presence of any bony dehiscences or fenestrations was also recorded.

The same operator (BDM) repeated all measurements at implant placement.

### 5.3. Secondary Variables

Keratinized mucosa width was measured after identifying the mucogingival junction (MGJ) with the roll-test, with a rotating movement of the periodontal probe (PCP-Unc 15, Hu-Friedy^®^, Chicago, Illinois, USA) by placing the tip at the MGJ and continuously adapting the probe’s axis on the curved surface of the gingiva up to the zenith of the alveolar ridge. All post-operative soft tissue assessments were conducted considering the position of the gingival margin (GM) at further follow-ups.

#### 5.3.1. Implant Stability

The maximum insertion torque at implant placement was recorded in Ncm by using a surgical motor with torque control (Implantmed, W&H, Bürmoos, Austria).

Furthermore, Resonance frequency analysis (RFA) was conducted to evaluate implant stability in the unit’s proprietary Implant Stability Quotient (ISQ) values (Osstell ISQ, Osstell AB, Goteborg, Sweden) at baseline and after one year.

#### 5.3.2. Marginal Bone Loss

Mesial and distal implant crestal bone levels were measured on standardized (Rinn Centratore XCP Evolution 2003, Dentsply, Rome, Italy) digital periapical X-rays for each implant obtained by using an imaging plate scanner (PSPIX²^®^, Acteon Group, Norwich, UK).

A calibrated software (SOPRO Imaging, Acteon Group, Norwich, UK) was used to estimate variations in the marginal peri-implant bone level. The implant length and width were used as references for calibration of measurements. 

The reference point for the bone level measurement was the implant shoulder. The bone level was digitally evaluated by measuring the distance between the implant shoulder and the first visible bone contact on the implant. 

#### 5.3.3. Peri-Implant Clinical Parameters

For each implant, the following clinical measurements were performed by using a periodontal probe (PCP-Unc 15, Hu-Friedy^®^, Chicago, Illinois, USA) with a light force (approximately 0.15 N), without anaesthesia, by the same previously trained calibrated examiner (BDM) at delivery of the prosthetic restorations and then after 6 and 12 months:

- Probing Pocket Depth (PPD). Measured in millimeters, is the distance from the mucosal margin to the bottom of the probable pocket
- Plaque Index (PI) recorded with dichotomic values (present/absent)
- Bleeding on probing recorded with dichotomic values (present/absent)

To achieve intra-examiner reliability, the examiner (BDM) was calibrated to show an agreement of 90% within 1 mm by duplicate measurements of probing depths on randomly selected teeth (10) and implants (10). 

### 5.4. Surgical Technique 

Standardized intraoral periapical X-rays were obtained prior to tooth extraction. 

One hour prior to surgery, prophylactic antibiotics were given to patients: 2 g of amoxicillin and clavulanic acid (Augmentin^®^, Roche S.p.A., Milan, Italy). At the beginning of the procedure, patients were instructed to rinse for one minute with 0.12% chlorhexidine gluconate (Curasept, Curaden Healthcare S.p.A, Saronno, Italy). All surgeries were performed under local anesthesia by the same operator (PP).

**Figure 1:**
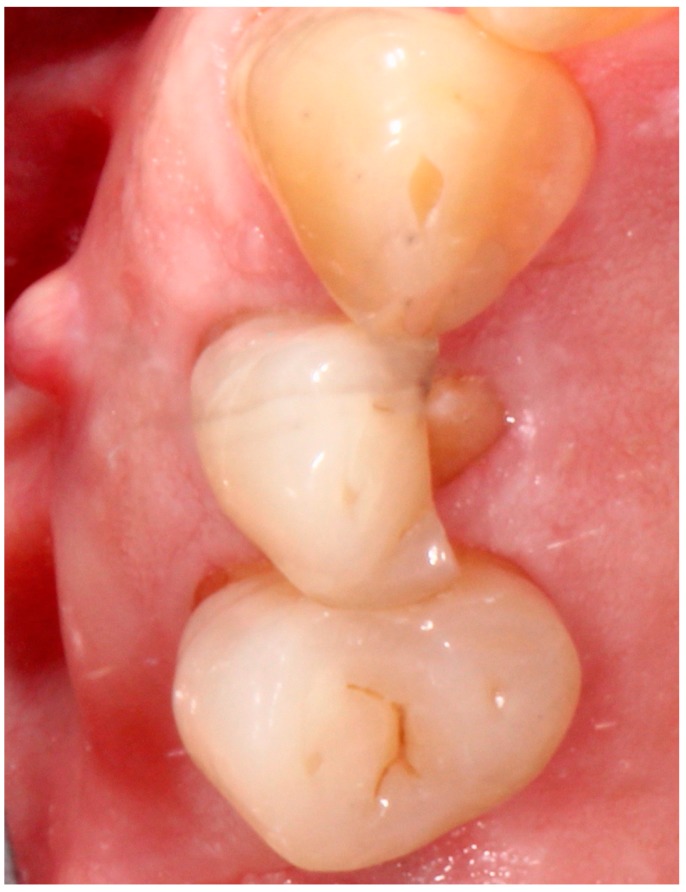
Clinical view of a 2.4 tooth presenting an endodontic failure with a buccal fistula.

**Figure 2:**
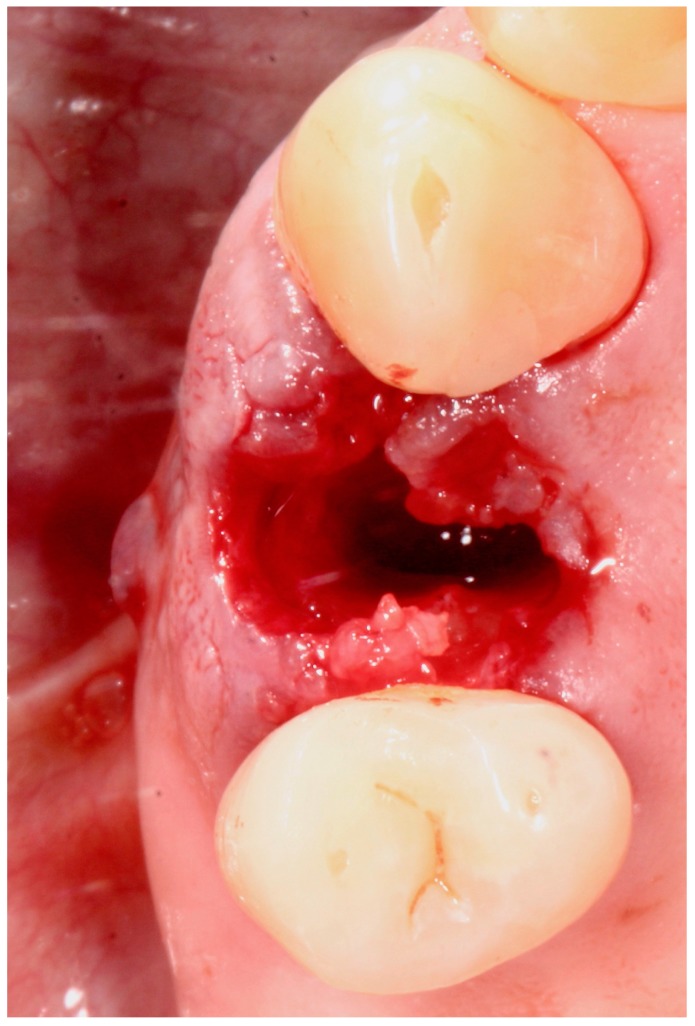
Alveolar cavity after atraumatic flapless extraction.

Atraumatic flapless extractions were performed by using periotomes, taking care to maintain the integrity of the facial bone wall (Figure 1 and Figure 2).

The residual walls of the alveolar cavity were carefully cleaned by using Lucas spoon and Gracey curettes and by rinsing the site abundantly with sterile saline solution 0.9%, in order to remove all granulation tissue. 

**Figure 3:**
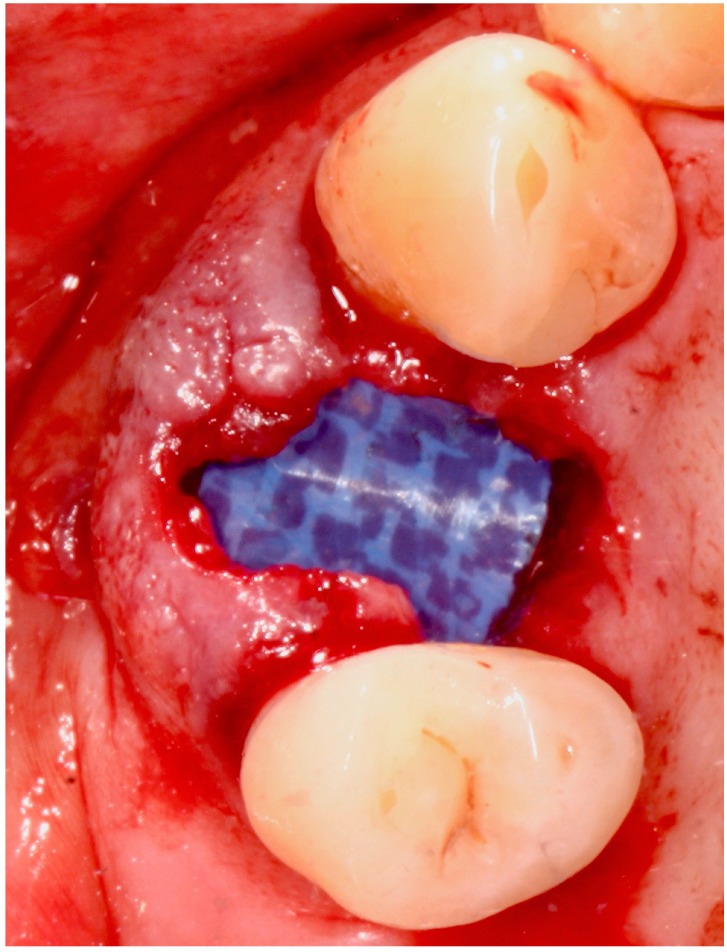
dense-polytetrafluoroethylene (d-PTFE) membrane placement.

Then, two small subperiosteal pockets on the buccal and palatal aspect of the alveolar cavity, extending 3 mm into the socket margins, were prepared by using a thin periotome, in order to allow stabilization of the membrane on the bony walls (Figure 3).

The d-ptfe membrane (Permamem, botiss biomaterials, Berlin, Germany) was adapted and placed to completely seal the socket. No biomaterial was used to graft the interior of the alveolar cavity.

**Figure 4:**
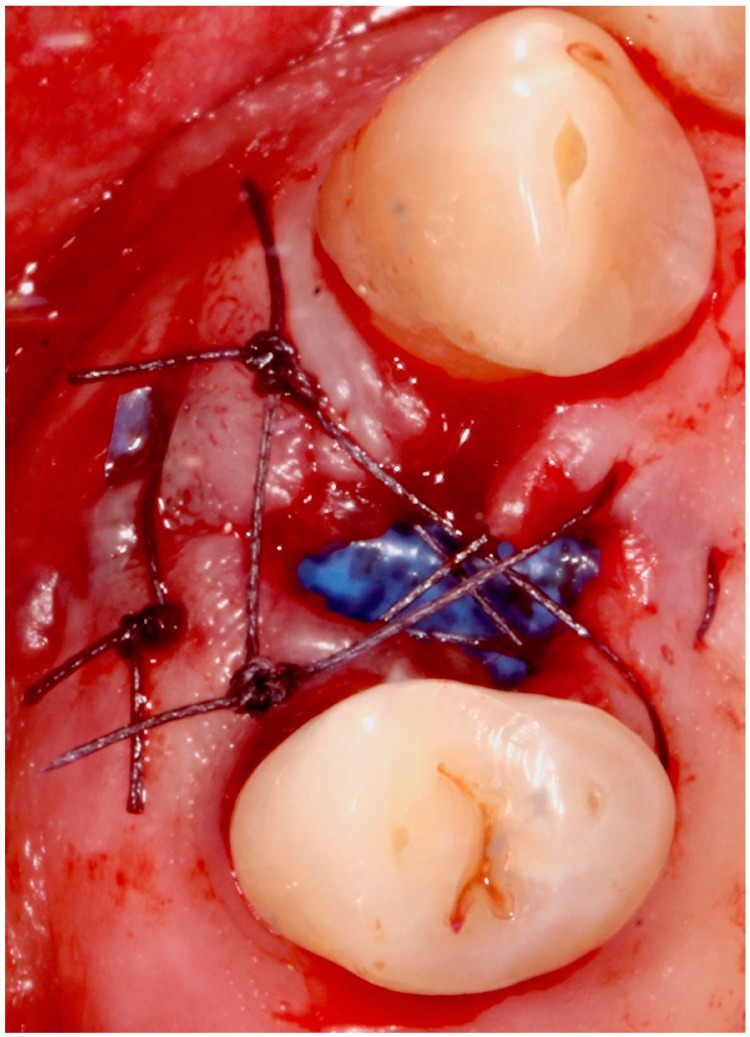
Membrane stabilization by sutures.

The membrane was stabilized with a horizontal mattress suture (Vycril 4.0, Ethicon, Johnson and Johnson, New Brunswick, NJ, USA) 3 mm below the most coronal part of the alveolar ridge and two cross single interrupted sutures (Figure 4).

Patients were instructed to rinse twice a day for one minute with chlorhexidine gluconate 0.2% (Curasept, Curaden Healthcare S.p.A, Saronno, Italy) and to avoid mechanical tooth cleaning at the surgical site for the first two weeks.

A soft diet was recommended and ibuprofen 600 mg (Brufen, Abbott, Verona, Italy) was prescribed to be taken as needed.

**Figure 5:**
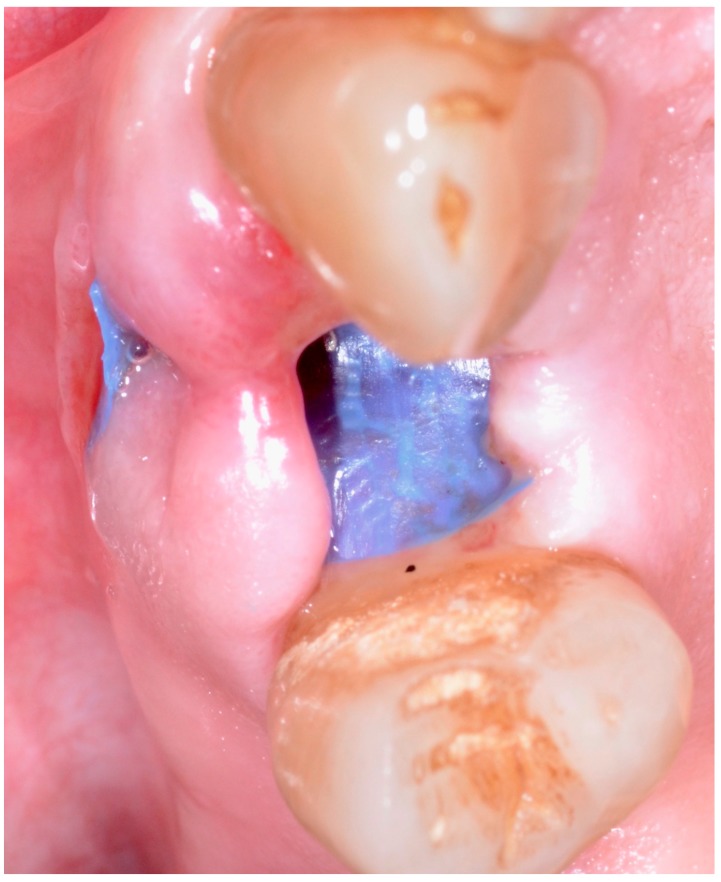
Clinical situation four weeks after membrane placement showing no signs of bacterial inflammation.

**Figure 6:**
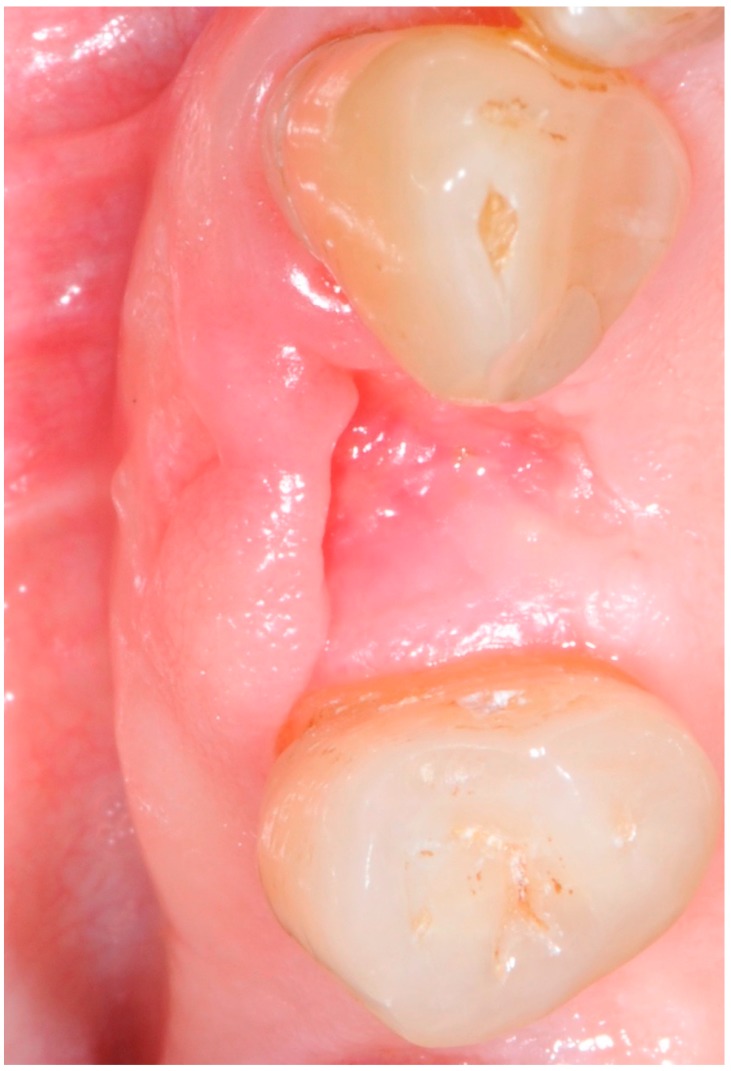
Clinical situation immediately after membrane removal.

Sutures were removed after 14 days, while non-surgical removal of the membrane was performed after four weeks without anesthesia by using tissue pliers (Figure 5 and Figure 6).

### 5.5. Implant Placement

Four months after the socket preservation procedure, a Cone Beam Computer Tomography (CBCT) of the surgical area was obtained and the appropriate dental implant was selected based on bone availability.

A mucoperiosteal flap was raised in the edentulous area and intrasulcular incisions were performed in the adjacent teeth, using a 15c scalpel blade (Hu-Friedy, Chicago, IL, USA).

**Figure 7:**
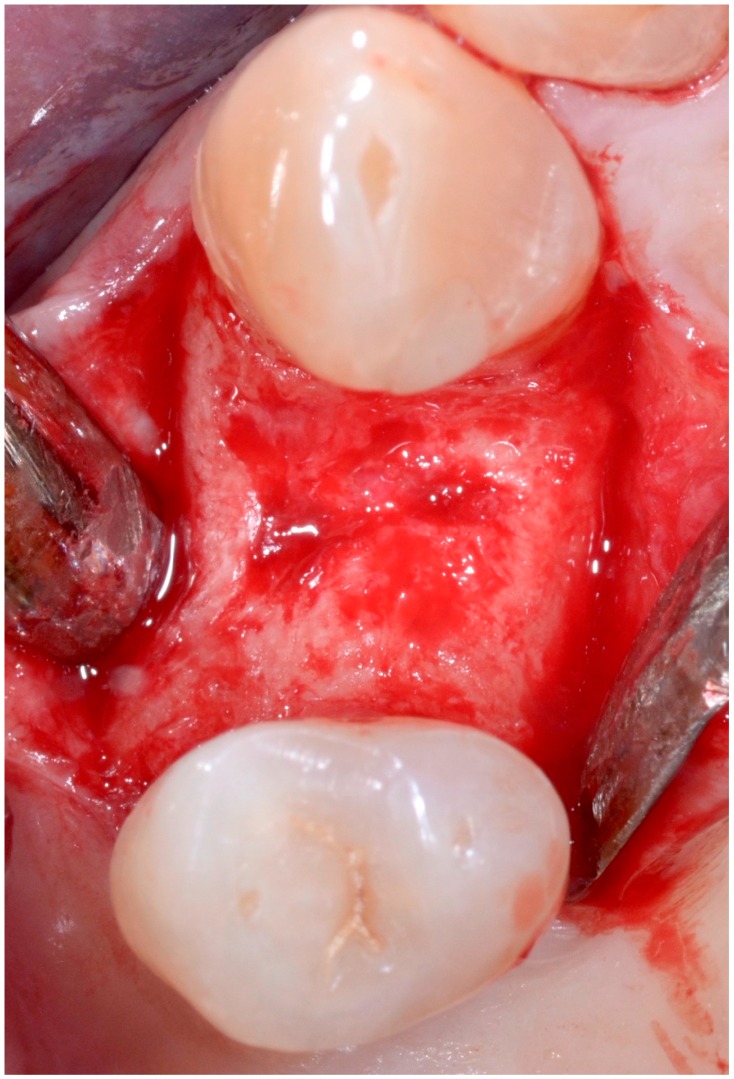
Alveolar ridge at surgical re-entry four months after tooth extraction.

**Figure 8:**
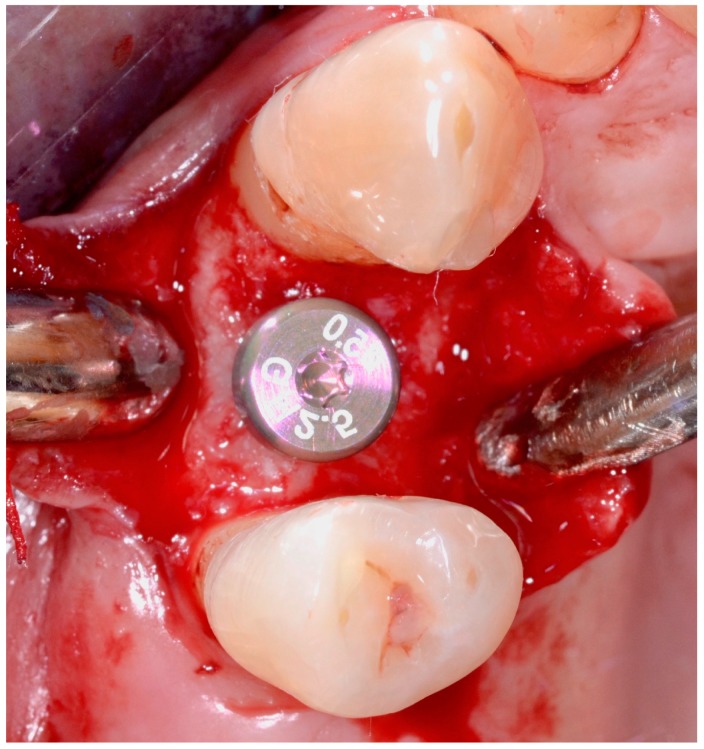
Implant placement.

Fully tapered titanium–zirconia dental implants with a chemically modified titanium surface (Blx, SLA Active Roxolid, Institut Straumann AG, Basel, Switzerland) were placed by following proper manufacturer’s instructions and the suggested drill protocol for medium bone density (Figure 7 and Figure 8).

**Figure 9:**
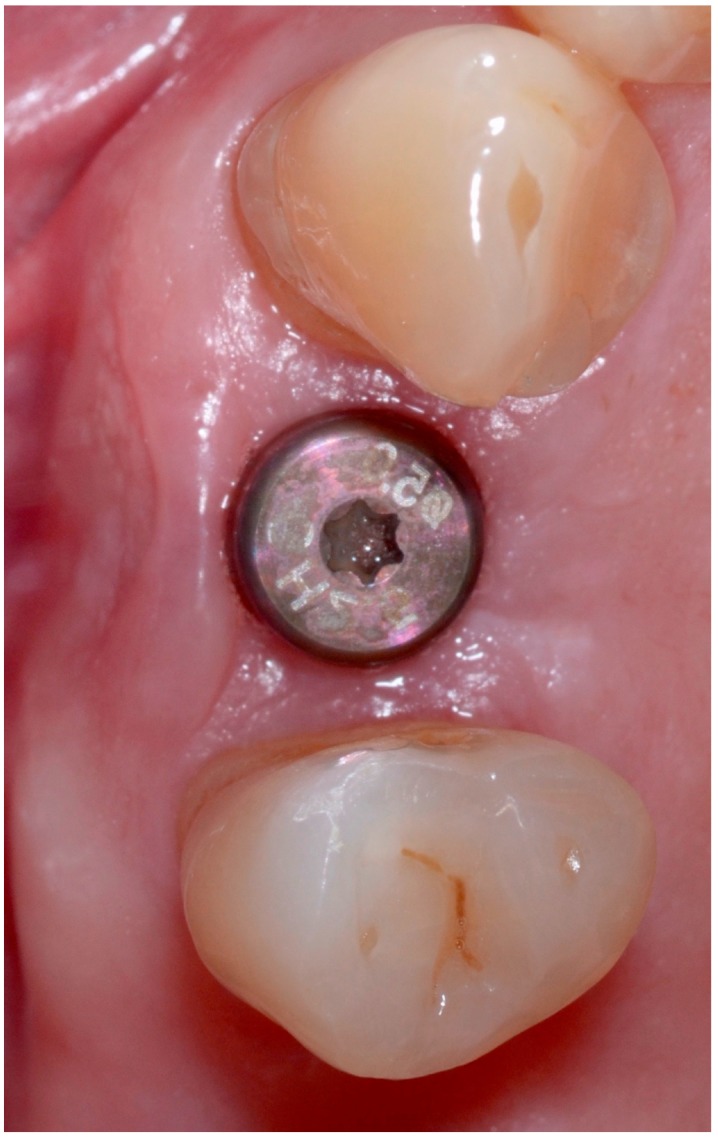
Clinical view at suture removal.

A transmucosal healing protocol was adopted by immediately placing healing abutments and closing the flap with interrupted sutures (Figure 9).

**Figure 10:**
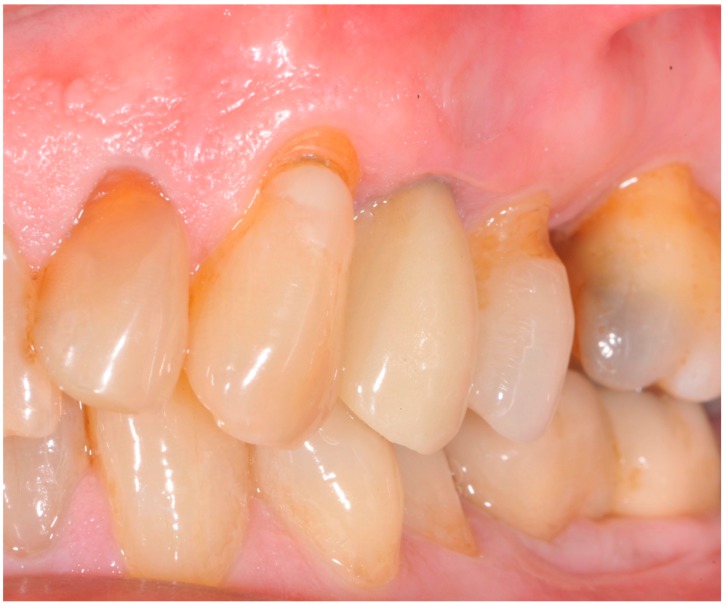
Clinical situation at delivery of the definitive screw-retained restoration.

Sutures were removed after 10 days and impressions were taken after four weeks using a polyether impression material (Impregum, 3M ESPE AG) with an open-tray using suitable impression copings to deliver definitive screw-retained gold-ceramic restorations (Figure 10).

### 5.6. Statistical Analysis

Data were evaluated using standard statistical analysis software (version 20.0, Statistical Package for the Social Sciences, IBM Corporation, Armonk, NY, USA).

Descriptive statistics was used to summarize data (mean, standard deviation) for each variable included. The Shapiro–Wilk test was used to determine whether or not the data conformed to a normal distribution. For intragroup comparison, the nonparametric Mann–Whitney U-test was used with a *p* value < 0.05 considered as statistically significant. 

## Figures and Tables

**Table 1:** Sample Demographics.

Study Variable	Descriptive Statistics
Sample size (*n*)	15
Male	9
Female	6
Age (y) ± SD (range)	49.4 ± 10.4 years (range: 35–67 years, median: 51 years)
Dental Implants BLX	
Diameter 4.5 mm	9
Diameter 3.75 mm	6
Length 10 mm	10
Length 12 mm	5

## References

[1] Pietrokovski J., Massler M. (1967). Alveolar ridge resorption following tooth extraction. J. Prosthet. Dent..

[2] Schropp L., Wenzel A., Kostopoulos L., Kar-ring T. (2003). Bone healing and soft tissue contour changes following single-tooth extraction: A clinical and radiographic 12-month prospective study. Int. J. Periodontics Restor. Dent..

[3] Araujo M.G., Sukekava F., Wennstrom J.L., Lindhe J. (2005). Ridge alterations following im-plant placement in fresh extraction sockets: An experimental study in the dog. J. Clin. Periodontol..

[4] Araújo M.G., Lindhe J. (2005). Dimensional ridge alterations following tooth extraction. An experimental study in the dog. J. Clin. Periodontol..

[5] Nevins M., Camelo M., De Paoli S., Friedland B., Schenk R., Parma-Benfenati S. (2006). A study of the fate of the buccal wall of extraction sockets of teeth with prominent roots. Prim. Dent. Care.

[6] Braut V., Bornstein M.M., Belser U., Buser D. (2011). Thickness of the anterior maxillary facial bone wall-a retrospective radiographic study using cone beam computed tomography. Int. J. Periodontics Restor. Dent..

[7] Chen S.T., Darby I. (2016). The relationship between facial bone wall defects and dimensional alterations of the ridge following flapless tooth extraction in the anterior maxilla. Clin. Oral Implant. Res..

[8] Januário A.L., Duarte W.R., Barriviera M., Mesti J.C., Araújo M.G., Lindhe J. (2011). Dimension of the facial bone wall in the anterior maxilla: A cone-beam computed tomography study. Clin. Oral Implant. Res..

[9] Chappuis V., Araújo M.G., Buser D. (2016). Clinical relevance of dimensional bone and soft tissue alterations post-extraction in esthetic sites. Periodontol. 2000.

[10] Irinakis T., Tabesh M. (2007). Preserving the Socket Dimensions With Bone Grafting in Single Sites: An Esthetic Surgical Approach When Planning Delayed Implant Placement. J. Oral Implant..

[11] Brügger O., Bornstein M.M., Kuchler U., Janner S., Chappuis V., Buser D. (2015). Implant Therapy in a Surgical Specialty Clinic: An Analysis of Patients, Indications, Surgical Procedures, Risk Factors, and Early Failures. Int. J. Oral Maxillofac. Implant..

[12] Khzam N., Arora H., Kim P., Fisher A., Mattheos N., Ivanovski S. (2015). Systematic Review of Soft Tissue Alterations and Esthetic Outcomes Following Immediate Implant Placement and Restoration of Single Implants in the Anterior Maxilla. J. Periodontol..

[13] Chappuis V., Engel O., Shahim K., Reyes M., Katsaros C., Buser D. (2015). Soft Tissue Alterations in Esthetic Postextraction Sites. J. Dent. Res..

[14] Thoma D.S., Naenni N., Figuero E., Hämmerle C., Schwarz F., Jung R.E., Sanz-Sánchez I. (2018). Effects of soft tissue augmentation procedures on peri-implant health or disease: A systematic review and meta-analysis. Clin. Oral Implant. Res..

[15] Perussolo J., De Souza A.B., Matarazzo F., De Oliveira R.P., Araújo M.G. (2018). Influence of the keratinized mucosa on the stability of peri-implant tissues and brushing discomfort: A 4-year follow-up study. Clin. Oral Implant. Res..

[16] Bonino F., Steffensen B., Natto Z.S., Hur Y., Holtzman L.P., Weber H.-P. (2018). Prospective study of the impact of peri-implant soft tissue properties on patient-reported and clinically assessed outcomes. J. Periodontol..

[17] Papi P., Pompa G. (2018). The Use of a Novel Porcine Derived Acellular Dermal Matrix (Mucoderm) in Peri-Implant Soft Tissue Augmentation: Preliminary Results of a Prospective Pilot Cohort Study. BioMed Res. Int..

[18] Mencio F., De Angelis F., Papi P., Rosella D., Pompa G., Di Carlo S. (2017). A randomized clinical trial about presence of pathogenic microflora and risk of peri-implantitis: Comparison of two different types of implant-abutment connections. Eur. Rev. Med. Pharmacol. Sci..

[19] Del Amo F.S.L., Lin G.-H., Monje A., Galindo-Moreno P., Wang H.-L. (2016). Influence of Soft Tissue Thickness on Peri-Implant Marginal Bone Loss: A Systematic Review and Meta-Analysis. J. Periodontol..

[20] De Angelis P., De Angelis S., Passarelli P.C., Liguori M.G., Manicone P.F., D’Addona A. (2019). Hard and Soft Tissue Evaluation of Different Socket Preservation Procedures Using Leukocyte and Platelet-Rich Fibrin: A Retrospective Clinical and Volumetric Analysis. J. Oral Maxillofac. Surg..

[21] Caiazzo A., Brugnami F., Mehra P. (2010). Buccal Plate Augmentation: A New Alternative to Socket Preservation. J. Oral Maxillofac. Surg..

[22] Jung R.E., Ioannidis A., Hämmerle C., Thoma D.S. (2018). Alveolar ridge preservation in the esthetic zone. Periodontol. 2000.

[23] Barone A., Toti P., Piattelli A., Iezzi G., Derchi G., Covani U. (2014). Extraction Socket Healing in Humans After Ridge Preservation Techniques: Comparison Between Flapless and Flapped Procedures in a Randomized Clinical Trial. J. Periodontol..

[24] Fickl S., Zuhr O., Wachtel H., Stappert C.F.J., Stein J.M., Hürzeler M.B. (2008). Dimensional changes of the alveolar ridge contour after different socket preservation techniques. J. Clin. Periodontol..

[25] Bassir S., Alhareky M., Wangsrimongkol B., Jia Y., Karimbux N. (2018). Systematic Review and Meta-Analysis of Hard Tissue Outcomes of Alveolar Ridge Preservation. Int. J. Oral Maxillofac. Implant..

[26] Avila-Ortiz G., Chambrone L., Vignoletti F. (2019). Effect of alveolar ridge preservation interventions following tooth extraction: A systematic review and meta-analysis. J. Clin. Periodontol..

[27] Del Fabbro M., Bucchi C., Lolato A., Corbella S., Testori T., Taschieri S. (2017). Healing of Postextraction Sockets Preserved With Autologous Platelet Concentrates. A Systematic Review and Meta-Analysis. J. Oral Maxillofac. Surg..

[28] Jonker B.P., Roeloffs M.W.K., Wolvius E.B., Pijpe J. (2016). The clinical value of membranes in bone augmentation procedures in oral implantology: A systematic review of randomised controlled trials. Eur. J. Oral Implant..

[29] Iocca O., Farcomeni A., Talib H.S., Pardiñas-Lopez S. (2016). Alveolar Ridge Preservation after tooth extraction: A Bayesian Network meta-analysis of grafting materials efficacy on prevention of bone height and width reduction. J. Clin. Periodontol..

[30] De Risi V., Clementini M., Vittorini G., Mannocci A., De Sanctis M. (2013). Alveolar ridge preservation techniques: A systematic review and meta-analysis of histological and histomorphometrical data. Clin. Oral Implant. Res..

[31] Macbeth N., Trullenque-Eriksson A., Donos N., Mardas N. (2016). Hard and soft tissue changes following alveolar ridge preservation: A systematic review. Clin. Oral Implant. Res..

[32] Kim S.-H., Kim -Y., Kim K.-H., Ku Y., Rhyu I.-C., Lee Y.-M. (2009). The efficacy of a double-layer collagen membrane technique for overlaying block grafts in a rabbit calvarium model. Clin. Oral Implant. Res..

[33] AlKanan A., Greenwell H., Patel A., Hill M., Shumway B., Lowy J. (2019). Ridge Preservation Comparing the Clinical and Histologic Healing of Membrane vs No-Membrane Approach to Buccal Overlay Grafting. Int. J. Periodontics Restor. Dent..

[34] Simion M., Baldoni M., Rossi P., Zaffe D. (1994). A comparative study of the effectiveness of e-PTFE membranes with and without early exposure during the healing period. Int. J. Periodontics Restor. Dent..

[35] Ronda M., Rebaudi A., Torelli L., Stacchi C. (2013). Expanded vs. dense polytetrafluoroethylene membranes in vertical ridge augmentation around dental implants: A prospective randomized controlled clinical trial. Clin. Oral Implant. Res..

[36] Ling L.-J., Hung S.-L., Lee C.-F., Chen Y.-T., Wu K.-M. (2003). The influence of membrane exposure on the outcomes of guided tissue regeneration: Clinical and microbiological aspects. J. Periodontal Res..

[37] Naung N.Y., Shehata E., Van Sickels J.E. (2019). Resorbable Versus Nonresorbable Membranes: When and Why?. Dent. Clin. N. Am..

[38] Soldatos N.K., Stylianou P., Koidou V.P., Angelov N., Yukna R., Romanos G.E. (2017). Limitations and options using resorbable versus nonresorbable membranes for successful guidedbone regeneration. Quintessence Int..

[39] Laurito D., Lollobrigida M., Gianno F., Bosco S., Lamazza L., De Biase A. (2017). Alveolar Ridge Preservation with nc-HA and d-PTFE Membrane: A Clinical, Histologic, and Histomorphometric Study. Int. J. Periodontics Restor. Dent..

[40] Hoffmann O., Bartee B.K., Beaumont C., Kasaj A., Deli G., Zafiropoulos G.-G. (2008). Alveolar Bone Preservation in Extraction Sockets Using Non-Resorbable dPTFE Membranes: A Retrospective Non-Randomized Study. J. Periodontol..

[41] Laurito D., Cugnetto R., Lollobrigida M., Guerra F., Vestri A., Gianno F., Bosco S., Lamazza L., De Biase A. (2016). Socket Preservation with d-PTFE Membrane: Histologic Analysis of the Newly Formed Matrix at Membrane Removal. Int. J. Periodontics Restor. Dent..

[42] Jambhekar S., Kernen F., Bidra A.S. (2015). Clinical and histologic outcomes of socket grafting after flapless tooth extraction: A systematic review of randomized controlled clinical trials. J. Prosthet. Dent..

[43] Avila-Ortiz G., Rodriguez J.C., Rudek I., Benavides E., Rios H., Wang H.-L. (2014). Effectiveness of three different alveolar ridge preservation techniques: A pilot randomized controlled trial. Int. J. Periodontics Restor. Dent..

[44] Mandarino D., Luz D., Moraschini V., Rodrigues D., Barboza E. (2018). Alveolar ridge preservation using a non-resorbable membrane: Randomized clinical trial with biomolecular analysis. Int. J. Oral Maxillofac. Surg..

[45] Barboza E.P., Stutz B., Mandarino D., Rodrigues D.M., Ferreira V.F. (2014). Evaluation of a Dense Polytetrafluoroethylene Membrane to Increase Keratinized Tissue. Implant. Dent..

[46] Deli G., Petrone V., De Risi V., Tadic D., Zafiropoulos G.-G. (2014). Longitudinal Implant Stability Measurements Based on Resonance Frequency Analysis After Placement in Healed or Regenerated Bone. J. Oral Implant..

[47] Malchiodi L., Balzani L., Cucchi A., Ghensi P., Nocini P. (2016). Primary and Secondary Stability of Implants in Postextraction and Healed Sites: A Randomized Controlled Clinical Trial. Int. J. Oral Maxillofac. Implant..

[48] Monje A., Ravidà A., Wang H.-L., A Helms J., Brunski J.B. (2019). Relationship Between Primary/Mechanical and Secondary/Biological Implant Stability. Int. J. Oral Maxillofac. Implant..

